# The decade of double-trouble: live birth and stillbirth sex ratio variation in Finland during the COVID-19 pandemic and Russia’s attack on Ukraine

**DOI:** 10.1007/s10654-026-01407-0

**Published:** 2026-05-27

**Authors:** Samuli Helle, Antti O. Tanskanen, Mirkka Danielsbacka

**Affiliations:** 1 INVEST Research Flagship Centre, University of Turku, Turku, Finland; 2 Department of Social Research, University of Turku, Turku, Finland; 3 Population Research Institute, Väestöliitto, Helsinki, Finland

**Keywords:** COVID-19, Exogenous shock, Sex ratio, War

## Abstract

Exogenous shocks experienced by the population like armed conflicts, natural disasters and economic downturns have all been suggested to correlate with variation in human sex ratio at birth, either through sex-specific embryonic mortality and/or primary sex adjustment. Recently experienced COVID-19 pandemic has also been related to changes in birth sex ratio in some countries but the results have been mixed. In the aftermath of the pandemic, Russia’s attack on Ukraine caused further emotional anxiety and economic hardship in Europe. This was also true in Finland, which has had a decade-long close trading history with its neighboring state, Russia. Here, we examined the influence of this “double trouble” on variation in proportion of males born alive as well as male proportion of stillbirths in Finland during 2000–2024, using high-quality monthly census panel data covering the entire Finnish population (a total of 1,355,037 live births and 4,096 stillbirths). The results from Bayesian aggregated binomial time-series regressions showed no evidence for COVID-19-related associations on either the proportion of male live births or stillbirths. We did find an increased proportion of males born alive after 4 months of Russia’s offensive but this finding should be considered as exploratory rather than confirmatory. If proportion of male live births or stillbirths can be regarded as a health indicator of the population, our results suggest that Finland as a society showed marked resilience in terms of non-responsive sex ratios when facing these two adverse exogenous shocks.

**Supplementary Information:**

The online version contains supplementary material available at https://doi.org/10.1007/s10654-026-01407-0.

## Introduction

At the population level, human sex ratio at birth, i.e., the proportion of males born [1], has been found to correlate with exogenous shocks, produced by e.g. wars [2], natural disasters as earthquakes, hurricanes and cyclones [3–5], ambient temperature [6, 7], famine [8] and economic adversity [9]. Commonly, but unequivocally, such shocks have been associated with a decline in proportion of males born [10]. Sex selection in utero as a mechanism has been put forward to explain these observations [11, 12] as male fetuses appear more fragile to poor developmental conditions than female fetuses [13]. Similarly, at the physiological level, higher preconception glucocorticoid hormone levels in women’s hair, reflecting chronic stress, have been suggested to influence newborn sex as more stressed mothers may deliver more likely daughters [14, 15]. Such findings have led to the suggestion that population birth sex ratios could serve as an indicator of population health [16, 17].

COVID-19 pandemic as an economic and health crisis had wide-ranging negative impacts on people’s everyday life and also affected monthly birth rates in countries world-wide [18]. Recent research has also explored the influence of the COVID-19 pandemic on the sex ratio at birth, revealing varying patterns across different regions (see [5] for a review). In England and Wales, a significant decrease in birth sex ratio was observed three months after the pandemic was declared in March 2020 but also a rise in birth sex ratio after nine months after the declaration of the pandemic [19]. Similar drop in birth sex ratio occurring three months after the onset of the pandemic was found in South Africa [20]. In Japan and Ireland, a lower birth sex ratio has instead been found nine months post-pandemic declaration [21, 22]. In contrast, studies conducted in Croatia and in the US found no statistically significant changes in birth sex ratio due to COVID-19 pandemic [23, 24]. In the US and Norway, selection against male twins in utero has been suggested to be responsible for a decline in birth sex ratio among twins born during the onset of the pandemic [25, 26]. A recent study of Mexico showed an overall constant increase in birth sex ratio during the pandemic at least until December 2022 [27]. These mixed findings underscore the potentially complex interplay between the COVID-19 pandemic and birth sex ratio, warranting more research on the topic.

The observed decreases in birth sex ratio three months later in some studies are thought to have been caused by maternal stress-related spontaneous male fetal loss during the second (13 to 27 weeks) or third trimester (28 to 40 weeks) of gestation [11, 12]. Such a mechanism has been considered adaptive for the mothers as evolutionary fitness-returns of males born during adverse conditions may be low compared to females [28, 29]. A mechanism for altered birth sex ratio 9 months following the exogenous shock might be a preference or selection against of conception of specific sex, perhaps owing to changes in sexual activity during the menstrual cycle [30]. While pregnant women might not have been more likely to get SARS-CoV-2 infections compared to non-pregnant women [31], recent studies have elucidated the impact of SARS-CoV-2 infection during pregnancy by showing that pregnant women with SARS-CoV-2 had increased risks of complications such as pre-eclampsia, gestational hypertension and higher rates of intensive care unit admissions [31, 32]. Risk of stillbirth, pre-term births and pre-labor caesarean births might also have been more common among the women who were admitted to hospital due to SARS-CoV-2 infection, who were unvaccinated and experienced moderate to severe symptoms compared to women with mild disease, although offspring sex-differences in these outcomes were not examined [33, 34]. These studies imply that in addition to psychological stress caused by the pandemic, it might have had a direct physiological impact on the pregnancy outcomes of the infected women. Therefore, it is also of interest to examine potential associations between external shocks and the sex ratio of stillborn children.

After the successful vaccination programs (for Finland, see [35]) and the less virulent variants that lessened the burden of ongoing pandemic on the society [36], Russia, Finland’s neighboring state with which it shares a 1,340 km border, launched its full-scale offensive against Ukraine in late February 2022. The attack, and the resulting geopolitical instability in Europe has increased psychological distress and uncertainty among Finns, who had fought against the Soviet Union during the World War II. The war triggered an energy crisis in Europe and economic sanctions against Russia that stifled Finland’s recovery from the COVID-19 pandemic through rising energy prices, increasing mortgage interest rates and inflation, worsening the economic growth of the country [37]. The economic consequences of this war that has now lasted more than 4 years on Finland’s economy are likely to outweigh those of shorter-lasted COVID-19 pandemic.

The aim of the current study is to examine whether the male proportion of live births and stillbirths varied according to two partly overlapping crises, the COVID-19 pandemic and the still ongoing Ukraine-Russia war, in Finland, covering the period from January 2000 to December 2024. Because the nature of such crises is likely to be complex in terms of their potential influences on the proportion of males born alive and stillborn males and previous examinations have produced mixed results, particularly in terms of the observed length of delay of the influence, we allowed for potential lags spanning up to 13 months after these shocks on the male proportion of live births and stillbirths. The 13-month delay is the maximum lag observed in previous studies so far [5]. To the best of our knowledge, the influence of Russia’s full-scale attack on Ukraine on the variation of the male proportion of live births and stillbirths has not been examined before.

## Methods

**Fig. 1:**
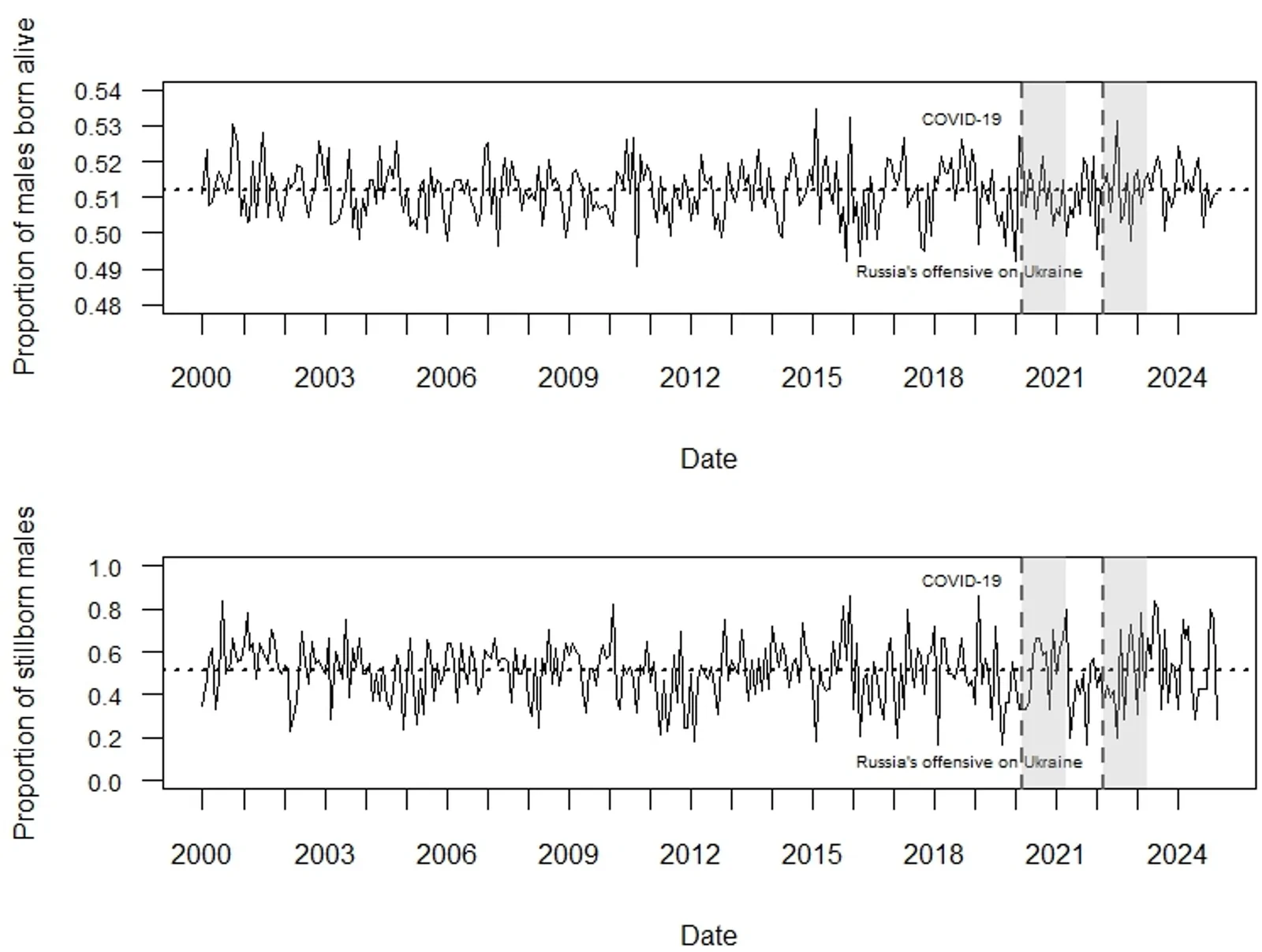
The proportion of male live births and stillbirths in Finland during 2000–2024. The dotted horizontal line depicts the mean of the series. Please note the differing scale of the y-axis. The grey shaded areas after the COVID-19 and Russia’s full-scale attack on Ukraine represent the 13-month lagged influences of these exogenous shocks

We retrieved monthly counts of male and female live births and stillbirths in Finland for the period January 2000 to December 2024 (THL Finnish Institute for Health and Welfare. Medical Birth Register. Unpublished data, Fig. 1). Stillbirth is defined as a fetus or a newborn who shows no signs of life at the time of birth after a pregnancy lasting at least 22 weeks or the newborn weighs at least 500 g. The selection of the starting date for the series is arbitrary and decided mainly on the basis to obtain a long enough series to accommodate the number of parameters estimated in the analyses (e.g., to avoid overfitting). The monthly live births ranged from 3,276 to 5,472 births whereas the monthly stillbirths ranged from 4 to 28 births. The mean proportion of male live births was 0.512% (SD = 0.08) and stillbirths was 0.510% (SD = 0.143). In total, our analyses included 1,355,037 live births and 4,096 stillbirths.

Unlike previous research on the topic, we do not model the proportion (or ratio) of males born using linear regression model but instead we directly model the number of males born (i.e., successes or events), given the number of total births (i.e., the number of trials) using aggregated binomial regression. This approach applying generalized linear model and logit link function is the recommended approach for analyzing sex ratios [1]. In other words, aggregated binomial regression automatically accommodates the varying monthly numbers of births while modelling the monthly numbers of males born or stillborn. This is especially important when analyzing the proportion of stillborn males where the monthly cases are very low and thus the outcome varies greatly from month to month (Fig. 1). The models fitted here included contemporary impact of the COVID-19 pandemic (3/2020, exact declaration date being 3/11/2020) and the full-scale start of the Ukraine-Russia war (3/2022; the full-scale offensive took place at 2/24/2022 but owing to the few days left in that month (i.e., four days) we expected the earliest response at March 2022) as well as their lagged impacts up to 13 months (i.e., distributed lag models were fitted). Note that the modelled impacts of these two shocks did not overlap in time (Fig. 1).

For these analyses, we applied Bayesian framework using the R package brms version 2.23 [38] for Bayesian multilevel modeling using Stan. To avoid flat priors on the logit scale that are not flat in the outcome probability scale [39] and to acknowledge that the effects on the proportion of live born and stillborn males are likely quite small, we used normal(0, 0.3) prior for the distributed lag regression parameters. In terms of odds ratios, this prior puts 95% probability density mass between the values of 0.56 and 1.80. Please note that an odds ratio of 2 would imply a predicted monthly proportion of male births of 0.667, which is clearly far greater than those observed during the whole study period. We thus regard this prior as regularizing or weakly informative, designed to avoid sampling of highly unlikely parameter space. For the intercept, we used normal(0, 0.4) prior, which has a mean of 0.5 on the proportion scale (i.e., zero on the logit scale), and the 95% probability density mass ranging from 0.31 to 0.69. The potential seasonality of monthly proportion of live born and stillborn males was accounted for by including a cyclic penalized cubic spline term into the model, which was parametrized as a random effect [40]. Potential long-term temporal fluctuations of the outcomes were allowed by including a thin plate spline for time in months since the start of the observation period (January 2000) (e.g., mathematical notation of the models fitted is given in the supplementary materials). The prior for the standard deviations of these smooth terms, as well as for the residual error, was half-Cauchy with a scale of 2. Note that proportion of live born and stillborn males are not correlated in this sample (bivariate Gaussian model for the monthly proportions of male live births and stillbirths, adjusting for seasonality and long-term fluctuations, residual correlation between proportion of live born and stillborn males (95% credibility intervals (CI): -0.06 (-0.18, 0.06)). Potential overdispersion of the binomial models was checked by fitting the models also using beta-binomial distribution but such models showed no improved predictive performance (Supplement Table S1).

The models were run for 10,000 iterations with a warm-up of 5,000 iterations and using four chains. Convergence of chains was inspected by checking the potential scale reduction factor [41], where potential scale reduction of 1.0 indicates full convergence, and visual inspection of the individual chains and their autocorrelation. Medians and their 95% credibility intervals were extracted for the parameters of interest from the corresponding posterior distributions. We further inspected the fit of the models with graphical posterior predictive checks (PPC). In PPCs, new datasets are generated according to the model and these data are then visually compared with the actual data used in fitting the model. If the model has performed well (in a sense of absolute fit to the data), generated and actual data will closely match each other. Here, all the scale reduction factors were effectively 1.00 and all the PPCs showed reasonable fit to the data (Supplement Table S2-S3 and Fig. S1). The residuals of fitted models were also checked for potential autocorrelation using autocorrelation (ACF) and partial autocorrelation plots (PACF).

## Results

**Fig. 2:**
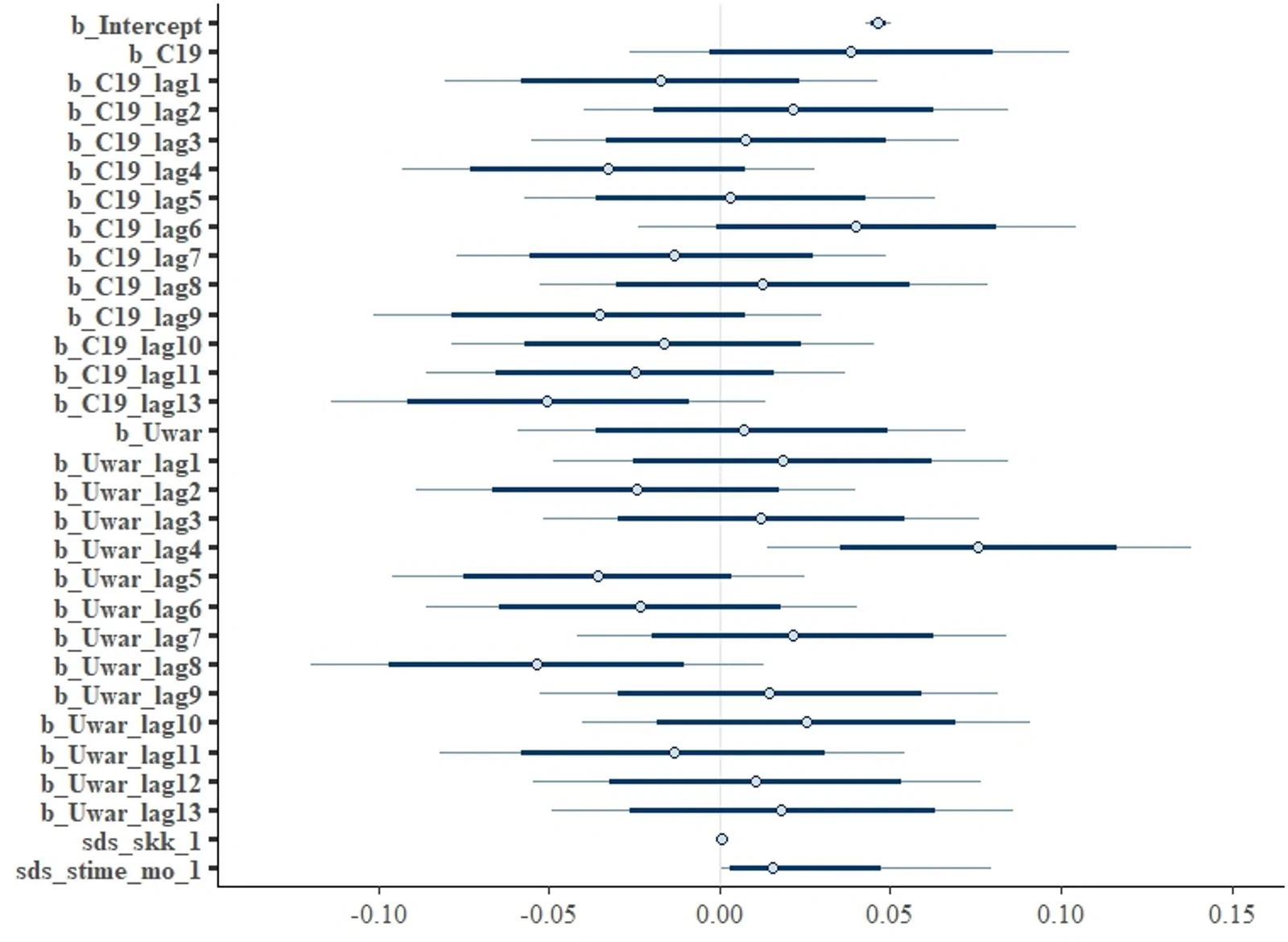
The results on logit scale for the proportion of male live births. The points represent the posterior median and the thick and thin segments show 80% and 95% intervals of the posterior distribution, respectively. Parameters “b_C19” represent the simultaneous and lagged influences of COVID-19 pandemic and parameters “b_Uwar” represent the same influences of Russia’s offensive against Ukraine on the proportion of male live births. Parameters “sds_skk_1” and “sds_stime_mo_1” represent the random effects for seasonality and long-term trend for the monthly sex ratio at birth in 2000–2024, respectively

None of the 13-month lags examined after the COVID-19 outbreak in March 2020 showed non-zero changes in the subsequent proportion of males born alive (Table S2; Fig. 2). Compared to the variation in the proportion of males born alive observed prior to the COVID-19, the proportion of live born males after the COVID-19 seemed rather stable, showing no large spikes in either direction (Fig. 1). With respect to the Russia’s full-scale offensive on Ukraine, we found a rise in the proportion of males born alive four months after the beginning of the war (Fig. 2): the odds of male live births increased by 7.9% (95% CI = 1.4–14.8%) in July 2022. This upward spike is clearly seen in Fig. 1, and it is one of the months with the highest proportion of males born alive in Finland during this century. The monthly proportion of males born showed weak seasonality, the proportion of male live births being the highest during the summer months (Supplement Fig. S2). Also, some long-term trend in the proportion of males born was found as the proportion of males born seemed to decrease from the year 2000 onwards and again rise after the year 2016 (Supplement Fig. S2). The residual autocorrelation plots of the fitted model showed two small spikes at the lags of 11 and 12 months, suggesting seasonality. (Supplement Fig. S3). However, our analysis already accounts for the potential 12-month seasonal component, making the relevance of these spikes suspicious. Because these single lags cannot be directly modelled in *brms* (i.e., using a subset AR(11,12) model), we modelled these manually as lagged predictors of monthly proportion of males born alive, and compared the predictive performance of this model to the original model. The results showed no improved predictive performance of the model including the lagged predictors of monthly proportion of males born alive (Supplement Fig. S3), and therefore our inference relied on the original, more parsimonious model.

**Fig. 3:**
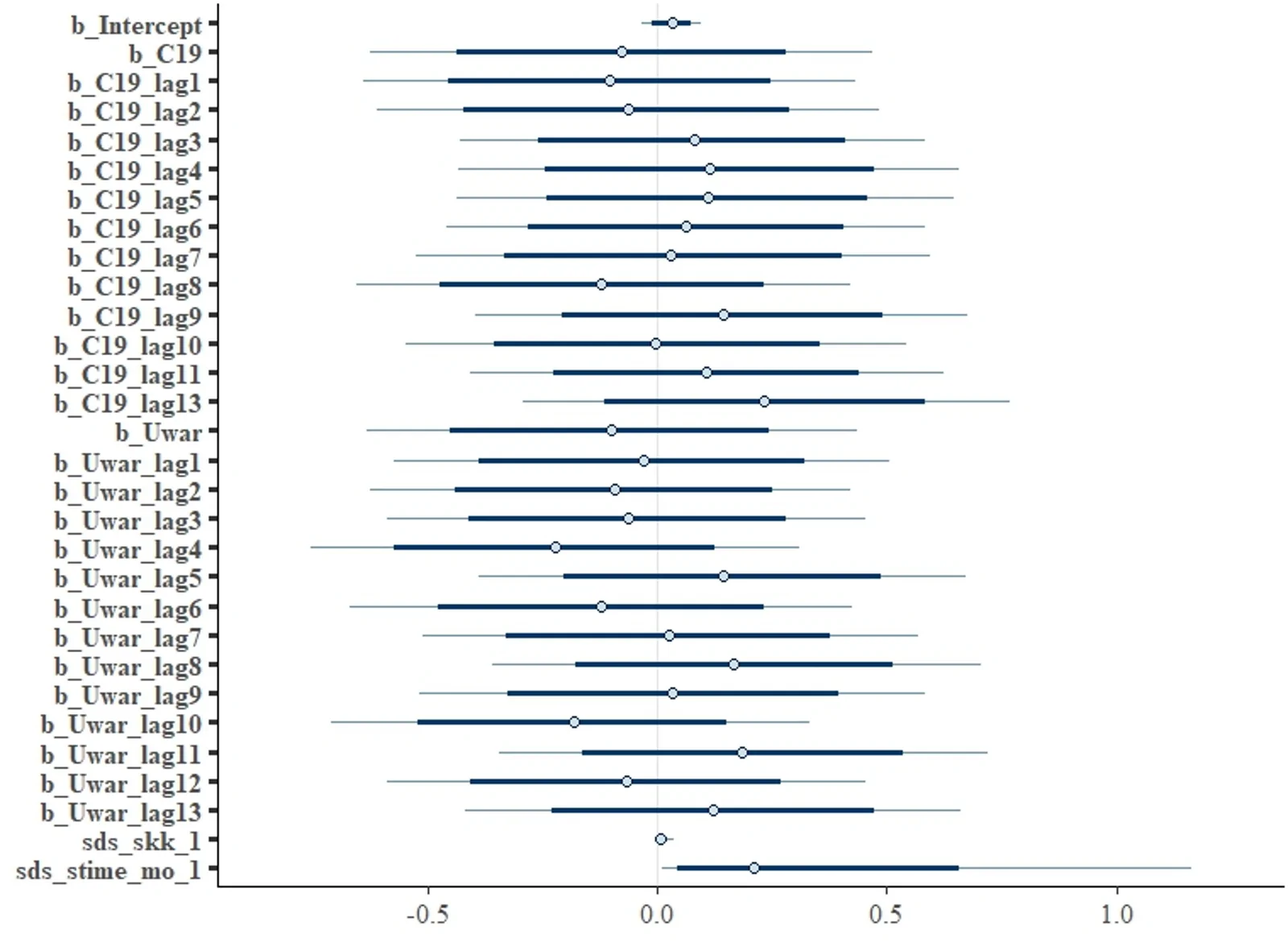
The results on logit scale for the proportion of male stillbirths. The points represent the posterior median and the thick and thin segments show 80% and 95% intervals of the posterior distribution, respectively. Parameters “b_C19” represent the simultaneous and lagged influences of COVID-19 pandemic and parameters “b_Uwar” represent the same influences of Russia’s offensive against Ukraine on the proportion of male stillbirths. Parameters “sds_skk_1” and “sds_stime_mo_1” represent the random effects for seasonality and long-term trend for the monthly sex ratio of stillborn children in 2000–2024, respectively

When looking at the monthly proportion of stillborn males, we found no non-zero regression coefficients with respect to the COVID-19 pandemic or the Russia’s war against Ukraine at any lag (Fig. 3). Again, the monthly proportion of stillborn males showed some evidence of seasonality, as the proportion of stillborn males was lowest around May-June and increased towards the end of the year (Supplement Fig. S4). The proportion of stillborn males also showed slight long-term curvature by being somewhat male-biased from the start of the study period but reaching equal proportion around the year 2012 (Supplement Fig. S4). Residual autocorrelation plots showed no autocorrelation at any lag (Supplement Fig. S5).

Moreover, when we considered the whole 13-month periods of the COVID-19 and the Russia’s attack on Ukraine instead of separate distributed lags, we find the same result: neither the COVID-19 period (Odds ratio (OR): 0.996, 95% CI = 0.978–1.015) nor the Russia’s attack on Ukraine (OR: 1.004, 95% CI = 0.985–1.024) were associated with changes in the proportion of males born alive. The same conclusion holds for the proportion of stillborn males as well (COVID-19 period, OR: 1.250, 95% CI = 0.910–1.705; Russia’s attack on Ukraine, OR: 0.944, 95% CI = 0.685–1.300). In a larger picture, the male proportion of live births (OR: 1.047, 95% CI = 0.808–1.345) or stillbirths (OR: 1.001, 95% CI = 0.982–1.018) did not shift after the start of the COVID-19 pandemic. Finally, the results were not qualitatively changed if we set our study period to begin from January 2010 apart from January 2000 as this starting point was purely arbitrary (results not shown).

Sensitivity analyses to assess the robustness of the results on the selected priors for the main models are given in the Supplement (Fig. S6-S7). It shows that our inference was robust to varying prior specifications.

## Discussion

Our results indicate that the start of the COVID-19 pandemic and the Russia’s full-scale attack on Ukraine were not commonly associated with the immediate or subsequent (up to delay of 13 months) changes in the male proportion of live births and stillbirths in Finland. Only in July 2022, roughly four months after the Russia’s offensive on Ukraine (beginning in the last days of February 2022), we found an increase in the proportion of male live births. Yet, a lag of four months is seldom reported in the literature after harmful exogenous shocks and particularly in favor of male births [5]. Hence, these findings do not suggest adverse impacts on the reproductive behavior of the Finnish population during these shocks. On the contrary, if related to the Russia-Ukraine war, this finding would imply a survival disadvantage of the commonly more robust female fetuses during the 2nd trimester of pregnancy after Russia’s offensive – we are not aware that such a phenomenon would have been suggested in the literature. On the other hand, the birth sex ratio peak in July 2022 coincides with the lifting of all COVID-19 restrictions for gathering in October 1st in 2021 (i.e., roughly 9–10 months earlier), along with effective vaccination progress, in Finland. This observation might be in line with the suggestion that short period of jubilation after WWII in allied countries, presumably accompanied with high coital rates influencing primary sex ratio, could have contributed to high post-war birth sex ratios in these countries [42]. Because to the best of our knowledge this was the first research to examine variation in the proportion of males born alive and stillborn males with respect to the Russia’s full-scale offensive on Ukraine, this might thus have been just a spurious temporal finding (i.e., not related to the onset of the war) as we examined (like many research before us) numerous lags without a clear theoretical rationale of their inclusion. Therefore, as other lags showed null findings, this single signal should be interpreted as exploratory and requiring replication.

Our results add to the mixed evidence on how the COVID-19 pandemic and other external stressors might have influenced proportion of males born. So far, COVID-19-related sex ratio bias at any lag have been reported in six countries [19–22, 27], whereas in two countries no such associations were found [23, 24]. One problematic issue is that there are no good candidates for exact mechanisms that would a priori predict the length of delay in birth sex ratio with respect to exogenous shocks – except for that the impact took place around the conception, i.e., there is association with a lag of roughly nine months. For example, in the case of natural disasters, although most studies suggest declines in the proportion of males born, the observed lag in the proportion of males born has found to vary from 3 to 11 months [3]. Similarly wide “effect window” has been documented in the case of the proportion of males born and diseases including the COVID-19 pandemic [5]. Although such shocks might in some cases be regarded to mimic natural experiments, it should be noted that causal interpretation of the results rests on largely untestable assumptions in any large population data (e.g., as-if random assignment). As long as this is true, the mixed results in the literature could simply owe to unmeasured (or residual) confounding that may vary from population to population, from time to time and from exogenous shock to another. This shortcoming concerns also the current study as we were unable to include any confounders into the analyses. Moreover, the current results on the shocks examined and the proportion of stillborn males should be regarded tentative as there were quite few monthly stillbirths in Finland during the study period.

Apart from the uncertainty of the reliability of the current evidence, we could speculate why the proportion of males born alive and stillborn in Finland seemed to be resilient against all the uncertainty related to the COVID-19 pandemic and the Russia’s offensive on Ukraine? With respect to the COVID-19, studies on birth trends during the pandemic in the Nordic countries has suggested that trust in institutions helped to combat pandemic-related uncertainty, and having even a short-term positive impact on fertility [43]. In Sweden, perceived uncertainties and trust in institutions were more important for fertility intentions than objective economic conditions. In Norway, the fertility increased during the pandemic particularly among those women who were economically most secure (e.g., higher-educated, aged of 28–35 years, having at least one prior child and those working in public administration) [44]. Also in Finland, birth rates showed a slight burst rather than drop during the COVID-19 pandemic [43, 18]. The finding that such elementary indicators of population fertility, monthly birth rate and monthly male proportion of live births and stillbirths, showed resilience to COVID-19 suggest that at least Finland as a nation succeeded to buffer the negative impacts of such unexpected exogenous shock on its population rather well. Or as like in Norway, the population fertility patterns observed during the study period may have been largely due to the fertility behavior of the economically less-affected part of the population. This could explain why we did not observe female-bias in birth sex ratio and/or male-bias in stillbirth sex ratio after the COVID-19. More research is needed on how Russia’s still ongoing aggression against Ukraine affects fertility behavior before we can draw any conclusions on the consequences of this crisis. Needless to say, Ukraine itself will be on the spotlight of such future research.

As already mentioned above, one potential shortcoming of the current study and prior literature is the nation-wide resolution of the data analysed. For example, we might have not expected the COVID-19 to had a concurrent influence on people across the whole country (e.g. urban population centers versus rural areas) and hence spatially more restricted or timed analyses might have given deeper insight how the pandemic influenced live birth and stillbirth sex ratios (e.g., by comparing temporal sex ratio dynamics at the same period but between “affected” and “unaffected” regions for causally more reliable estimates, see e.g. [45]. and “synthetic control method” of [46]). Similar heterogeneity might be expected based on e.g. people’s occupation, ethnicity and socio-economic status as the pandemic hit people differently based on these factors [47]. For instance, ethnic minorities in the US took proportionally greater toll in hospitalization and mortality than the more privileged white people [48]. More importantly, because the mechanisms producing such population-level phenomena originate from the physiological level within individuals, the most convincing evidence for these ideas should also come from the individual-level data. In support for this [14] and [15], have used women’s hair to measure their glucocorticoid hormone levels, reflecting chronic (preconception) stress, and associated it with the sex of the children born. Therefore, most of the research on the topic, relying merely on “associations with time” likely fall short on directly capturing individual-level stress in women. The reported associations, including the current findings, are hence likely plagued by measurement error in the predictor variables (i.e., intervention of the event or time because the event has non-perfect correlation with true stress experienced by the women in the population), which is known to produce biased inference of the true effects (e.g. [49]). Given this, and the fact that it is usually hard to control for potential individual-level confounders using population-level data (not to mention potential unmeasured confounding), it is not surprising that literature has been so mixed.

Closely related to the potential heterogeneity issue mentioned above, is the underlying evolutionary rationale commonly used to frame these studies: The Trivers-Willard hypothesis (TWH [28]). In short, the TWH predicts that women should bias sex allocation towards female offspring in times of adversity. In addition to the still-ongoing debate of the existence of the TWH in humans at the individual level after decades of research [e.g., 50–54], in evolutionary biology, it has long been acknowledged that applying the TWH to population-level phenomena is problematic [55] and this particularly has hampered the research of human sex ratios [10]. This is because the TWH is about relative sex allocation between women in a population: women in relatively poor condition compared to other women in the population should bias sex allocation towards daughters whereas women in relatively good condition should prefer to invest in sons. Such a mechanism might be at work at the individual level, but almost any population-level pattern, depending on the proportion of women truly affected by exogenous adversity, could emerge [55]. And as discussed in the previous paragraph, it is highly unlikely that economic hardship, psychological stress and health issues related to the COVID-19 and Russia’s offensive on Ukraine war affected all the women in the country equally. Furthermore, the potential maternal stress-induced sex determination is unlikely the sole agent influencing offspring sex, increasing the difficulties to interpret population sex ratio variation [55]. For example, major crises like world wars have had the opposite effect on the birth sex ratios than smaller-scale and shorter crises [56]. Therefore, we urge researcher to be cautious when interpreting such results as evidence for or against of the TWH. The same problem may also underlie the reliable use of population birth sex ratio as an indicator of population health [16].

In conclusion, our analyses, based on high-quality register data and advanced methods, suggest that the onset of COVID-19 and Russia’s attack on Ukraine had minimal influence on the proportion of male live births and stillbirths in Finland. These findings may speak of the resilience of the Finnish society in terms of fertility outcomes against such external adversities that did have psychological, health and economic impacts on the society. It should however be noted that this conclusion may not apply to the less privileged subgroups in the population and that mechanistic interpretation of these results remains speculative. Despite of this, the real reproductive consequences of these external factors may not have manifested themselves on the sex ratios but on the later performance of the children born during the most stressing times of these crises. Searching for such potential long-term sex-specific influences in women from differing socio-economic strata would provide much stronger evidence for or against the TWH as the current population-level examinations.

## Supplementary Information

Below is the link to the electronic supplementary material.

Supplementary Material 1

## Data Availability

The data we used in this study can be requested from Finnish Institute for Health and Welfare.
