# Supplementary material for: The decade of double-trouble: live birth and stillbirth sex ratio variation in Finland during the COVID-19 pandemic and Russia’s attack on Ukraine
Source: Eur J Epidemiol. 2026 May 27;41(8):1019–28. doi: 10.1007/s10654-026-01407-0 (PMC13612614; doi:10.1007/s10654-026-01407-0)
Supplement: Supplementary file 1 [file 10654_2026_1407_MOESM1_ESM.docx]

**Online supplement for Helle et al. “The decade of double-trouble: Live birth and stillbirth sex ratio variation in Finland during the COVID-19 pandemic and Russia’s attack on Ukraine”**

*Mathematical notation of the models fitted*

Mathematical notation of the models used to examine monthly variation in the proportion of male live births and stillbirths according to the contemporary impact of COVID-19 and the full-scale onsett of the Ukraine-Russia war as well as their lagged impacts up to 13 months.

Indexing: *t* = 1, . . . ,*T* indexes months; *m_t_* ∈ {1, . . . , 12} is calendar month; *u_t_* ≥ 0 is months since January 2000.

$$\begin{aligned} t\in\{1, \ldots, T\}\#\left( 1 \right) \end{aligned}$$

$$\begin{aligned} m_{t}\in\{1, \ldots, 12\}\#\left( 2 \right) \end{aligned}$$

$$\begin{aligned} u_{t}\in\mathbb{R}_{\backslash\geq0.}\#\left( 3 \right) \end{aligned}$$

Data and parameters: *y_t_* is the number of male births / stillbirths; *n_t_* is the total births / stillbirths; *p_t_* is the probability that a birth / stillbirth is male in month *t*.

$$\begin{aligned} y_{t}\in\{0, 1, \ldots, n_{t}\}\#\left( 4 \right) \end{aligned}$$

$$\begin{aligned} y_{t}\sim\text{Binomial}\left( n_{t}, p_{t} \right).\#\left( 5 \right) \end{aligned}$$

Link: the binomial model uses a logit link, defining the linear predictor *η_t_*.

$$\begin{aligned} \text{logit}\left( p_{t} \right)= \eta_{t}.\#\left( 6 \right) \end{aligned}$$

Linear predictor: contemporaneous influence and the lags up to 13 months for COVID-19 (C19) and the Russia-Ukraine (U) war and seasonal and long-run smooth terms.

$$\begin{aligned} \eta_{t}= \alpha+\sum_{k=0}^{13} \beta_{k}^{\left( C \right)}C_{t,k}+\sum_{k=0}^{13} \beta_{k}^{\left( U \right)}U_{t,k}+f_{season}\left( m_{t} \right)+f_{trend}\left( u_{t} \right)\#\left( 7 \right) \end{aligned}$$

Pulse indicators: *t*_C19_ and *t*_U_ mark the onset months; the lag blocks do not overlap.

$$\begin{aligned} C_{t,k}\mathbb{= I}\{t=t_{C19}+k\}, \#\left( 8 \right) \end{aligned}$$

$$\begin{aligned} U_{t,k}\mathbb{= I}\{t=t_{U}+k\}, k = 0, 1, . . . , 13.\#\left( 9 \right) \end{aligned}$$

Smooth terms: *f*_season_ is a cyclic penalized cubic spline in *m_t_* (parameterized as a random effect); *f*_trend_ is a thin-plate spline in *u_t_*.	(10)

In plain-text summary, the fitted model was (in the case of proportion of male live births) as follows:

Outcome:
y_t = males_born (number of male live births), with trials n_t = total_born (total number of live births)

Likelihood:
y_t ~ Binomial(n_t, p_t)

Link function:
logit(p_t) = η_t

Linear predictor:
η_t = β0
+ Σ_{k=0}^{13} β_C19,k * C19_{t−k}
+ Σ_{k=0}^{13} β_Uwar,k * Uwar_{t−k}
+ f1(month)
+ f2(time)

Smooth terms:
f1(month): cyclic cubic spline with k = 12
f2(time): smooth function of continuous time

*The brms code used to fit the models were as follows (given only for the proportion of male live births):*

model <- bf(males_born | trials(total_born) ~

C19+C19_lag1+C19_lag2+C19_lag3+C19_lag4+C19_lag5+C19_lag6+C19_lag7+C19_lag8+C19_lag9+C19_lag10+C19_lag11+C19_lag12+C19_lag13+Uwar+Uwar_lag1+Uwar_lag2+Uwar_lag3+Uwar_lag4+Uwar_lag5+Uwar_lag6+Uwar_lag7+Uwar_lag8+Uwar_lag9+Uwar_lag10+Uwar_lag11+Uwar_lag12+Uwar_lag13+s(month, bs='cc', k=12)+s(time_month))

prs <- c(set_prior("normal(0,0.3)", class = "b"),

set_prior("cauchy(0,2)", class = "sds"),

set_prior("normal(0,0.4)", class = "Intercept"))

m1<-brm(model,

family = binomial(),

data = data,

chains = 4, iter = 10000, seed = 321,

backend = "cmdstanr",

prior=prs,

control = list(adapt_delta = 0.999))

These models took 585.5 and 319.3 seconds to run with respect to live birth and stillbirth sex ratios, respectively, on a 32GB RAM and Intel i7-11850H 2.5GHz computer. Software and session metadata used to estimate these models is given below.

R version: R version 4.5.0 (2025-04-11 ucrt)

brms version: 2.23.0

cmdstanr version: 0.9.0

CmdStan version: 2.36.0

*Overdispersion: comparison of binomial and beta-binomial models*

In order to evaluate potential overdispersion in the models fitted, we compared our binomial models to beta-binomial models that allow for overdispersion (i.e., estimating the precision parameter “phi” in brms). Because we are not expecting to see strong overdispersion (see the PPC-plots in Fig 1 below), we used the gamma(1, 0.01) prior for the parameter “phi”. The comparison was based on the predictive performance of binomial and beta-binomial models, conducted by approximating the leave-one-out cross-validation (LOO-CV) using Pareto smoothed importance sampling (PSIS) (the *loo* package in R; Vehtari et al. 2017). The PSIS-LOO-CV computes the expected log predictive density (ELPD) for each model and provides a relative measure of how well a model predicts new data, taking into account the uncertainty in the model parameters (Vehtari et al. 2017). A higher ELPD value indicates a better-performing model, and the difference in ELPD between models and the associated standard error allows an assessment of the statistical significance of the difference in their predictive performance (Vehtari et al. 2017). A raw rule of thumb is that, if the sample size is not small, the ELPD difference (elpd_diff) should clearly be more than twice as large as the standard error of ELPD difference (se_diff) to indicate differing predictive performance of the models compared. In all the models evaluated, Pareto *k* estimates showed good reliability (*k* < 0.7). Table S1 shows that with respect to both the proportion of male live birth and stillbirths, the beta-binomial model allowing for overdispersion showed poorer predictive performance compared to binomial model (this was far more obvious in the case of proportion of male live births). Furthermore, dispersion parameters (with 95% credibility intervals, CI) for binomial models further supported the conclusion that the data shows only trivially more variance than the binomial assumes, so the extra parameter in the beta-binomial is not justified - it adds complexity without improving fit. We therefore retained the binomial specification.

**Table S1** Results of PSIS-LOO-CV comparisons.

|  |  | Elpd | Elpd_diff | SE_diff | Dispersion (95% CI) |
| --- | --- | --- | --- | --- | --- |
| SRB | |  |  |  |  |
|  | Binomial | -1527.0 | 0.0 | 0.0 | 1.20 (1.15, 1.26) |
|  | Beta-binomial | -1560.4 | -33.3 | 7.3 |  |
| SRS | |  |  |  |  |
|  | Binomial | -595.1 | 0.0 | 0.0 | 1.05 (1.02, 1.10) |
|  | Beta-binomial | -595.7 | -0.6 | 0.9 |  |

Vehtari, A., Gelman, A., & Gabry, J. (2017). Practical Bayesian model evaluation using leave-one-out cross-validation and WAIC. Statistics and Computing, 27(5), 1413-1432. <https://doi.org/10.1007/s11222-016-9696-4>

**Table S2** Results of Bayesian aggregated binomial regression model for the monthly proportion of male live births in Finland 2000-20024. For all the parameters estimated, posterior median and their 95% credibility intervals (CI) are given. “C19” stands for COVID-19 pandemic and “Uwar” stands for Russia’s attack on Ukraine, with their corresponding lagged influences. PSR denotes to potential scale reduction factor of the chains. Bulk and tail effective sample sizes, Bulk_ESS and Tail_ESS, respectively, reflect the sampling efficiency of the bulk and tails of the posterior distributions of the parameters. Random effects give the standard deviation of the corresponding parameters.

|  |  |  | Lower | Higher |  |  |  | Probability |
| --- | --- | --- | --- | --- | --- | --- | --- | --- |
|  |  | Median | 95% CI | 95% CI | PSR | Bulk_ESS | Tail_ESS | of direction |
| Fixed effects | |  |  |  |  |  |  |  |
|  | Intercept | 0.0465 | 0.0428 | 0.0502 | 1.0003 | 43210 | 14771 | 100.0 |
|  | C19 | 0.0384 | -0.0262 | 0.1024 | 1.0005 | 39588 | 14436 | 88.4 |
|  | C19_lag1 | -0.0174 | -0.0803 | 0.0464 | 1.0002 | 38441 | 14595 | 69.6 |
|  | C19_lag2 | 0.0217 | -0.0395 | 0.0843 | 1.0000 | 43398 | 14121 | 74.1 |
|  | C19_lag3 | 0.0078 | -0.0549 | 0.0701 | 1.0004 | 38860 | 14002 | 58.9 |
|  | C19_lag4 | -0.0329 | -0.0930 | 0.0279 | 1.0002 | 40702 | 14723 | 85.5 |
|  | C19_lag5 | 0.0031 | -0.0571 | 0.0632 | 1.0002 | 40205 | 14623 | 54.1 |
|  | C19_lag6 | 0.0401 | -0.0238 | 0.1044 | 1.0002 | 38591 | 13653 | 89.7 |
|  | C19_lag7 | -0.0137 | -0.0770 | 0.0488 | 1.0006 | 38996 | 13538 | 66.5 |
|  | C19_lag8 | 0.0128 | -0.0528 | 0.0786 | 1.0002 | 43078 | 14049 | 64.6 |
|  | C19_lag9 | -0.0354 | -0.1013 | 0.0297 | 1.0004 | 43827 | 14043 | 85.2 |
|  | C19_lag10 | -0.0163 | -0.0784 | 0.0451 | 1.0007 | 40807 | 14181 | 70.1 |
|  | C19_lag11 | -0.0248 | -0.0860 | 0.0370 | 1.0001 | 40191 | 15338 | 77.9 |
|  | C19_lag12 | 0.0128 | -0.0472 | 0.0728 | 1.0006 | 39923 | 13216 | 66.1 |
|  | C19_lag13 | -0.0504 | -0.1138 | 0.0136 | 1.0008 | 40675 | 13632 | 94.4 |
|  | Uwar | 0.0067 | -0.0592 | 0.0724 | 1.0009 | 42808 | 14087 | 58.3 |
|  | Uwar_lag1 | 0.0185 | -0.0488 | 0.0845 | 1.0002 | 38442 | 13940 | 71.1 |
|  | Uwar_lag2 | -0.0245 | -0.0888 | 0.0401 | 1.0000 | 38815 | 13635 | 77.8 |
|  | Uwar_lag3 | 0.0121 | -0.0515 | 0.0762 | 0.9999 | 39656 | 13970 | 65.0 |
|  | Uwar_lag4 | 0.0758 | 0.0140 | 0.1383 | 1.0002 | 40797 | 15133 | 99.2 |
|  | Uwar_lag5 | -0.0356 | -0.0958 | 0.0250 | 1.0005 | 41557 | 14650 | 87.5 |
|  | Uwar_lag6 | -0.0231 | -0.0857 | 0.0402 | 1.0002 | 39016 | 13540 | 76.4 |
|  | Uwar_lag7 | 0.0213 | -0.0415 | 0.0839 | 1.0009 | 40148 | 13562 | 74.8 |
|  | Uwar_lag8 | -0.0535 | -0.1198 | 0.0128 | 1.0004 | 38238 | 14959 | 93.8 |
|  | Uwar_lag9 | 0.0146 | -0.0525 | 0.0814 | 1.0004 | 40911 | 14064 | 67.1 |
|  | Uwar_lag10 | 0.0254 | -0.0401 | 0.0912 | 1.0004 | 39713 | 13714 | 77.5 |
|  | Uwar_lag11 | -0.0134 | -0.0820 | 0.0542 | 1.0005 | 42547 | 14064 | 65.1 |
|  | Uwar_lag12 | 0.0107 | -0.0548 | 0.0766 | 1.0004 | 42089 | 15203 | 62.6 |
|  | Uwar_lag13 | 0.0183 | -0.0492 | 0.0863 | 1.0002 | 39263 | 14220 | 70.3 |
|  | linear trend | -0.0108 | -0.1184 | 0.0884 | 1.0007 | 12975 | 10469 | 62.9 |
| Random effects | |  |  |  |  |  |  |  |
|  | sds_seasonality | 0.0005 | 0.00002 | 0.0017 | 1.0004 | 7512 | 9812 |  |
|  | sds_trend | 0.0215 | 0.0008 | 0.0796 | 1.0002 | 6516 | 9973 |  |

**Table S3** Results of Bayesian aggregated binomial regression model for the monthly proportion of male stillbirths in Finland 2000-20024. For all the parameters estimated, posterior median and their 95% credibility intervals (CI) are given. “C19” stands for COVID-19 pandemic and “Uwar” stands for Russia’s attack on Ukraine, with their corresponding lagged influences. PSR denotes to potential scale reduction factor of the chains. Bulk and tail effective sample sizes, Bulk_ESS and Tail_ESS, respectively, reflect the sampling efficiency of the bulk and tails of the posterior distributions of the parameters. Random effects give the standard deviation of the corresponding parameters.

|  |  |  | Lower | Higher |  |  |  | Probability |
| --- | --- | --- | --- | --- | --- | --- | --- | --- |
|  |  | Median | 95% CI | 95% CI | PSR | Bulk_ESS | Tail_ESS | of direction |
| Fixed effects | |  |  |  |  |  |  |  |
|  | Intercept | 0.0315 | -0.0324 | 0.0952 | 1.0004 | 31861 | 14020 | 83.6 |
|  | C19 | -0.0763 | -0.6251 | 0.4702 | 1.0001 | 31709 | 13673 | 60.4 |
|  | C19_lag1 | -0.1036 | -0.6418 | 0.4309 | 1.0000 | 33044 | 13706 | 65.1 |
|  | C19_lag2 | -0.0651 | -0.6087 | 0.4818 | 1.0005 | 32728 | 14817 | 59.5 |
|  | C19_lag3 | 0.0776 | -0.4298 | 0.5837 | 1.0003 | 30775 | 13407 | 61.9 |
|  | C19_lag4 | 0.1147 | -0.4326 | 0.6565 | 1.0001 | 32552 | 15309 | 65.7 |
|  | C19_lag5 | 0.1088 | -0.4368 | 0.6460 | 1.0002 | 31994 | 14325 | 65.6 |
|  | C19_lag6 | 0.0629 | -0.4600 | 0.5840 | 1.0003 | 32910 | 14468 | 59.5 |
|  | C19_lag7 | 0.0331 | -0.5269 | 0.5932 | 1.0001 | 34240 | 13844 | 55.4 |
|  | C19_lag8 | -0.1195 | -0.6533 | 0.4212 | 1.0002 | 31503 | 14904 | 67.4 |
|  | C19_lag9 | 0.1413 | -0.3968 | 0.6753 | 1.0000 | 33125 | 14506 | 69.7 |
|  | C19_lag10 | -0.0038 | -0.5472 | 0.5413 | 1.0000 | 30531 | 14356 | 50.7 |
|  | C19_lag11 | 0.1072 | -0.4058 | 0.6230 | 1.0002 | 35531 | 15545 | 66.1 |
|  | C19_lag12 | 0.1525 | -0.3654 | 0.6749 | 1.0005 | 33383 | 15039 | 71.0 |
|  | C19_lag13 | 0.2339 | -0.2927 | 0.7668 | 1.0003 | 34433 | 15185 | 79.9 |
|  | Uwar | -0.1014 | -0.6311 | 0.4374 | 1.0004 | 33934 | 14732 | 64.5 |
|  | Uwar_lag1 | -0.0314 | -0.5752 | 0.5041 | 1.0002 | 33445 | 14847 | 54.5 |
|  | Uwar_lag2 | -0.0940 | -0.6234 | 0.4199 | 1.0007 | 32432 | 14704 | 63.6 |
|  | Uwar_lag3 | -0.0658 | -0.5876 | 0.4559 | 1.0002 | 33262 | 14884 | 59.9 |
|  | Uwar_lag4 | -0.2216 | -0.7548 | 0.3097 | 1.0000 | 35672 | 12773 | 79.6 |
|  | Uwar_lag5 | 0.1432 | -0.3891 | 0.6705 | 1.0005 | 34520 | 14985 | 69.8 |
|  | Uwar_lag6 | -0.1238 | -0.6706 | 0.4227 | 1.0004 | 32661 | 15585 | 66.2 |
|  | Uwar_lag7 | 0.0239 | -0.5113 | 0.5672 | 1.0002 | 35925 | 14365 | 53.5 |
|  | Uwar_lag8 | 0.1665 | -0.3599 | 0.7040 | 1.0001 | 32670 | 14909 | 73.3 |
|  | Uwar_lag9 | 0.0328 | -0.5173 | 0.5850 | 1.0003 | 33321 | 14675 | 55.5 |
|  | Uwar_lag10 | -0.1849 | -0.7109 | 0.3334 | 1.0004 | 33751 | 14240 | 75.5 |
|  | Uwar_lag11 | 0.1864 | -0.3448 | 0.7208 | 1.0002 | 32866 | 14782 | 74.3 |
|  | Uwar_lag12 | -0.0669 | -0.5872 | 0.4546 | 1.0001 | 33322 | 14949 | 60.7 |
|  | Uwar_lag13 | 0.1209 | -0.4179 | 0.6603 | 1.0002 | 33364 | 14931 | 66.9 |
|  | linear trend | -0.0926 | -0.5761 | 0.4408 | 1.0002 | 18969 | 15833 | 65.1 |
| Random effects | |  |  |  |  |  |  |  |
|  | sds_seasonality | 0.0102 | 0.0004 | 0.0361 | 1.0001 | 5986 | 8487 |  |
|  | sds_trend | 0.3053 | 0.0108 | 1.1639 | 1.0005 | 6268 | 8732 |  |

**Fig. S1** Posterior predictive checks (PPC) for the models predicting the proportion of males born alive and stillborn males. Blue lines represent simulated data sets (n = 100 for each case) from the model fitted, and the black line represents the original data (i.e., observations). In both cases, reasonably close match between blue lines and the black line suggest that the model fitted adequately to the data.

**Fig. S2** Estimated seasonality (month) and long-term trend (time_month) of the proportion males born in Finland 2000-2024.

**Fig. S3** Autocorrelation function (ACF) and partial autocorrelation function (PACF) plots of model residuals predicting the proportion of males born. The dotted lines represent approximate 95% confidence bounds for the hypothesis of no autocorrelation in residuals. There are small spikes at the lag of 11 and 12 months, suggesting seasonality. However, as our analysis already accounts for the 12-month seasonal component of birth sex ratio variation, these spikes are unexpected and may be spurious. When we added those spikes into the model manually as lagged predictors of monthly proportion of males born alive, the original model and the model with the additional lags did not differ in their predictive performance (PSIS-LOO-CV: elpd difference = -2.6 with s.e. of the difference = 3.5; using the "two standard error rule": 7.0 > 2.6, meaning that the models are equally good at predicting the outcome).

**Fig. S4** Estimated seasonality (month) and long-term trend (time_month) of the proportion of stillborn males in Finland 2000-2024.

**Fig. S5** Autocorrelation function (ACF) and partial autocorrelation function (PACF) plots of model residuals predicting stillbirth sex ratio. The dotted lines represent approximate 95% confidence bounds for the hypothesis of no autocorrelation in residuals.

**Fig. S6** Prior sensitivity analysis for the model predicting the proportion of male live births in Finland 2000-2024. 95% intervals of the posterior distributions are shown. Three alternative prior settings were examined:

*Original Prior Specification for the model parameters*

*β* ∼ 𝒩 (0, 0.3), for all fixed effect coefficients

α ∼ 𝒩 (0, 0.4), for the intercept

σ_smooth_ ∼ Cauchy (0, 2), for standard deviations of smooth spline terms

#### Alternative 1: Less informative Priors

*β* ∼ 𝒩 (0, 1), for all fixed effect coefficients

α ∼ 𝒩 (0, 1), for the intercept

σ_smooth_ ∼ Cauchy (0, 5), for standard deviations of smooth spline terms

#### Alternative 2: More informative Priors

*β* ∼ 𝒩 (0, 0.1), for all fixed effect coefficients

α ∼ 𝒩 (0, 0.2), for the intercept

σ_smooth_ ∼ Cauchy (0, 1), for standard deviations of smooth spline terms

**Fig. S7** Prior sensitivity analysis for the model predicting the proportion of stillborn males in Finland 2000-2024. 95% intervals of the posterior distributions are shown. The prior specifications are as above.
